# Multiple roles of branched-chain amino acid metabolism in tumour progression

**DOI:** 10.1186/s12929-025-01132-y

**Published:** 2025-04-09

**Authors:** Lin Wang, Feng Shi, Ya Cao, Longlong Xie

**Affiliations:** 1 Key Laboratory of Carcinogenesis and Cancer Invasion of Chinese Ministry of Education, XiangYa Hospital, Central South University, Changsha, 410078 China; 2 Department of Radiology, The Affiliated Children’s Hospital of Xiangya School of Medicine (Hunan Children’s Hospital), Central South University, Changsha, 410078 China; 3 Key Laboratory of Carcinogenesis of National Health Commission, Cancer Research Institute and School of Basic Medical Science, Xiangya School of Medicine, Central South University, Changsha, 410078 China

**Keywords:** BCAA metabolism, Metabolic reprogramming, Tumour progression, Tumour resistance, Tumour immunity, mTORC signalling pathway

## Abstract

Metabolic reprogramming enables tumour cells to sustain their continuous proliferation and adapt to the ever-changing microenvironment. Branched-chain amino acids (BCAAs) and their metabolites are involved in intracellular protein synthesis and catabolism, signal transduction, epigenetic modifications, and the maintenance of oxidative homeostasis. Alterations in BCAA metabolism can influence the progression of various tumours. However, how BCAA metabolism is dysregulated differs among depending on tumour type; for example, it can manifest as decreased BCAA metabolism leading to BCAA accumulation, or as enhanced BCAA uptake and increased catabolism. In this review, we describe the role of BCAA metabolism in the progression of different tumours. As well as discuss how BCAA metabolic reprogramming drives tumour therapy resistance and evasion of the antitumour immune response, and how these pro-cancer effects are achieved in part by activating the mTORC signalling pathway. In-depth investigations into the potential mechanisms by which BCAA metabolic reprogramming affects tumorigenesis and tumour progression can enhance our understanding of the relationship between metabolism and cancer and provide new strategies for cancer therapy.

## Introduction

The initiation and progression of tumours depend on the reprogramming of cellular energy metabolism [1]. The phenomenon of metabolic reprogramming in tumorigenesis can be attributed to the need for rapid growth and adaptation of tumour cells to the immune microenvironment. First, tumour cells are characterized by continuous growth, unlimited proliferation, and invasion into normal tissues. To meet their ever-increasing nutritional and biosynthetic demands, the metabolic pathways providing energy and other molecules to tumour cells are adaptively adjusted [2]. Second, tumour cells often need to adapt to hypoxic environments, maintain redox homeostasis, and evade immune surveillance. Metabolic reprogramming assists tumour cells in adapting to their dynamic microenvironment to sustain survival. Although disruption of glucose metabolism, represented by the Warburg effect, is the main feature of metabolic reprogramming [3], altered amino acid metabolism, lipid metabolism, and nucleotide metabolism also have crucial functions in tumour development [4].

Amino acids are essential for cellular survival, serving as the raw materials for protein synthesis and providing a source of energy and metabolites. Amino acid metabolism is promoted in tumour cells, as the breakdown of these molecules provides both nitrogen and carbon to meet the requirements for rapid growth [5]. Branched-chain amino acids (BCAAs), a class of essential amino acids, include leucine, isoleucine, and valine. They are recognized as critical components that support the survival, growth, proliferation, migration, and invasion of tumour cells. Alterations in BCAA metabolism can influence various tumour phenotypes and serve as markers for assessing tumour prognosis [6, 7]. Diverse tumour types or the same tumour under different conditions exhibit variations in the demand for BCAA metabolism, which is dependent primarily on the intensity of BCAA catabolism. This is reflected by changes in circulating BCAA levels and the activity and expression of related enzymes [8–10]. In addition, dysregulation of BCAA metabolism drives tumour drug resistance, immune escape or antitumour immunity and is an important feature of tumorigenesis and progression [11–15]. This review focuses on the role of BCAA metabolism reprogramming in the development of different tumours and how it drives anti-tumor immune response and treatment resistance, primarily in cancer types with high morbidity and mortality rates, including lung cancer [10, 11, 16], liver cancer [17–19], breast cancer (BC) [15, 20–23], colorectal cancer (CRC) [12, 24–27], leukaemia [28–34], glioblastoma [35–39], pancreatic ductal adenocarcinoma [40–44], and ovarian cancer [9, 45, 46].

## Branched-chain amino acid metabolic network and its reprogramming in tumour cells

Branched-chain amino acids (BCAAs), comprising leucine (Leu), isoleucine (Ile), and valine (Val), are a class of essential amino acids that cannot be synthesized by mammals themselves and must therefore be acquired through dietary intake and protein degradation [47]. Under physiological conditions, precisely maintaining the balance between the intake and consumption of branched-chain amino acids is crucial at both the cellular and systemic physiological levels; this balance involves nutrient absorption, metabolic regulation, and associated biochemical pathways [48]. Imbalances in the intake and/or breakdown of BCAA metabolism may lead to deficiencies or excessive accumulation, thereby predisposing individuals to various diseases, including diabetes [49, 50], heart failure [51, 52], and cancer [53]. Hence, precise regulation of this balance is crucial for maintaining the normal physiological state of animals and ensuring proper biological functions.

### The intake of BCAAs

The L-type amino acid transporter (LAT) family of transmembrane transport proteins serves as the main pathway for BCAA entry into the cytoplasm. The LAT family consists of four neutral amino acid transporters, namely, LAT1 (SLC7A5), LAT2 (SLC7A8), LAT3 (SLC43A1), and LAT4 (SLC43A2). Among them, LAT1 is the main BCAA transporter and plays a key role in the reprogramming of BCAA metabolism in tumour cells [54]. LAT1 is the major amino acid transporter that supports the development of hepatocellular carcinoma (HCC). Knockdown of LAT1 attenuates BCAA transport activity and significantly reduces HCC cell proliferation [53]. In addition, high expression of LAT1 in breast cancer cells increases leucine uptake, leading to tamoxifen resistance in patients and significantly shortening their survival time [20, 55]. Furthermore, the overexpression of G protein-coupled receptor family C group 5 member C (GPRC5C) activates the NF-κB-LAT1 axis, which increases the concentration of circulating BCAAs and enhances BCAA catabolism, thereby increasing the energy supply through the tricarboxylic acid (TCA) cycle. This ultimately leads to increased invasiveness of acute myeloid leukaemia (AML) cells [34]. Notably, the LAT1 inhibitor JPH203 has progressed into phase I clinical trials [56]; it specifically targets LAT1 to inhibit the proliferation of advanced solid tumours and improve prognosis [56, 57].

### BCAA catabolism

**Fig. 1:**
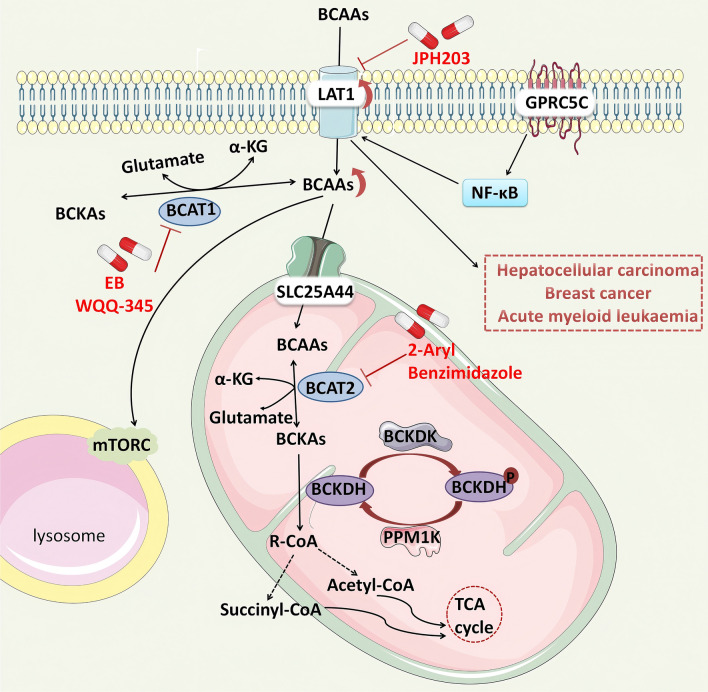
BCAA metabolism and tumours. BCAAs (leucine, isoleucine, valine) are transported into the cell by LAT1 and catalysed by BCAT1 to produce BCKAs in the cytoplasm. After entering the mitochondria through the SLC25A44 transporter, BCKAs can be catalysed by BCAT2 for transamination and then undergo irreversible oxidative decarboxylation to generate branchchain acyl coenzymes through BCKDH, and then generate acetyl-CoA and succinyl-coenzyme A through a series of pathways to enter the TCA cycle for functional production. The activity of BCKDH is regulated by BCKDK and PPM1K. The phosphorylation of BCKDH by BCKDK inhibits BCKDH activity, whereas PPM1K dephosphorylates BCKDH and activates BCKDH. Elevated levels of LAT1-mediated BCAAs lead to the proliferation of HCC cells and drug resistance in breast cancer patients. GPRC5C increases the aggressiveness of AML through the NF-κB-LAT1. The LAT1 inhibitor JPH203.The BCAT1 inhibitor EB and WQQ-345. The BCAT2 inhibitor 2-Aryl Benzimidazole

The catabolic metabolism of BCAAs primarily comprises two major steps, namely, BCAA transamination and branched-chain keto acid oxidation decarboxylation. Branched-chain aminotransferases (BCATs) reversibly transfer α-amino groups from BCAAs to α-ketoglutaric acid (α-KG), generating the corresponding branched-chain α-keto acids (BCKAs) and glutamate. BCATs include two isoenzymes: branched-chain aminotransferase 1 (BCAT1), which is present in the cytosol and is predominantly expressed in limited tissues such as the brain and kidneys, and mitochondrial branched-chain aminotransferase 2 (BCAT2), which is widely expressed in various tissues except for the liver [58]. In the cytoplasm, BCAAs are used for protein synthesis or reversible transamination under the catalysis of BCATs. However, after entering the mitochondria via SLC25A44, BCKAs produced by BCAAs can be further oxidized to the end products acetyl-CoA and/or succinyl-CoA to enter the tricarboxylic acid cycle, in addition to being re-ammoniated [59]. The Branched-chain alpha-ketoate dehydrogenase (BCKDH) complex, located in the inner mitochondrial membrane, is the first key rate-limiting enzyme in BCAA catabolism and irreversibly decarboxylates branched-chain alpha-ketoate dehydrogenase (BCKDH). Notably, the BCKDH enzyme consists of three subunits (E1, E2, and E3) [58], of which the E1 subunit is a decarboxylase encoded by the BCKDHA (E1α) and BCKDHB genes [48] and is capable of catalysing the oxidative decarboxylation of BCKAs to produce the corresponding acyl intermediate while releasing carbon dioxide. The activity of the BCKDH complex is regulated by posttranslational covalent modifications involving the phosphorylation of the E1α subunit. BCKD kinase (BCKDK) phosphorylates the E1α subunit of BCKDHA, leading to its inactivation. Conversely, the mitochondrial-targeted protein phosphatase Mg^2+^ and Mn^2+^-dependent 1K (PPM1K) then dephosphorylates the E1α subunit of BCKDHA to reactivate the complex [60] (Fig. 1).

### BCAA metabolism and other essential amino acids

Essential amino acids not only serve as fundamental building blocks for constructing substances necessary for life but also function as signalling molecules capable of initiating biosynthesis or participating in the regulation of cellular life processes. For example, methionine is involved primarily in biological processes such as polyamine biosynthesis, DNA methylation, and the formation of glutathione [61]. Dysregulation of tryptophan metabolism mainly promotes tumour growth and immune evasion by suppressing the tumour immune microenvironment [62]. Threonine is an important substrate for the tRNA modification enzyme YrdC N(6)-threonylcarbamoyl transferase domain containing (YRDC), and it promotes the self-renewal ability of glioblastoma stem cells by maintaining a high rate of translation [63]. BCAAs are the second largest nitrogen source in cells after glutamine, accounting for 35% of the essential amino acids in muscle protein and 50% in food. Studies have shown that BCAAs are essential for maintaining the survival of HCC cells under glutamine deprivation conditions [17]. BCAAs and their metabolites can act as signalling cofactors to alter the epigenome, regulate the cellular redox state, and affect immune cell function. However, in contrast to other essential amino acids in tumours, BCAAs activate tmainly the mammalian target of the rapamycin (mTOR) signalling pathway to exert a series of oncogenic effects.

## Mechanisms by which BCAA metabolic reprogramming mediates tumour progression

To date, numerous studies have elucidated the associations between disruptions in BCAA metabolism and various phenotypes in tumours, including lung cancer [10, 11, 16], hepatocellular carcinoma [17–19, 64], breast cancer [15, 20–23], colorectal cancer (CRC) [12, 24–27], leukaemia [28–34], glioblastoma [35–39], pancreatic ductal adenocarcinoma [40–44], and ovarian cancer [9, 45, 46]. However, how BCAA metabolism is dysregulated differs depending on tumour types. Different tumours exhibit distinct metabolic patterns, which are primarily classified into two categories: (1) decreased BCAA metabolism leads to the accumulation of BCAAs, which in turn activates the mTOR signalling pathway [22, 65–67] and (2) enhanced BCAA uptake and catabolism provides intermediates for other pathways and mediates epigenetic regulation [66–68]. The core feature of BCAA metabolism dysregulation is the abnormal activation of metabolic enzymes/pathways/metabolites within the BCAA catabolism pathway.

### BCAA hypocatabolism promotes tumour development

**Fig. 2:**
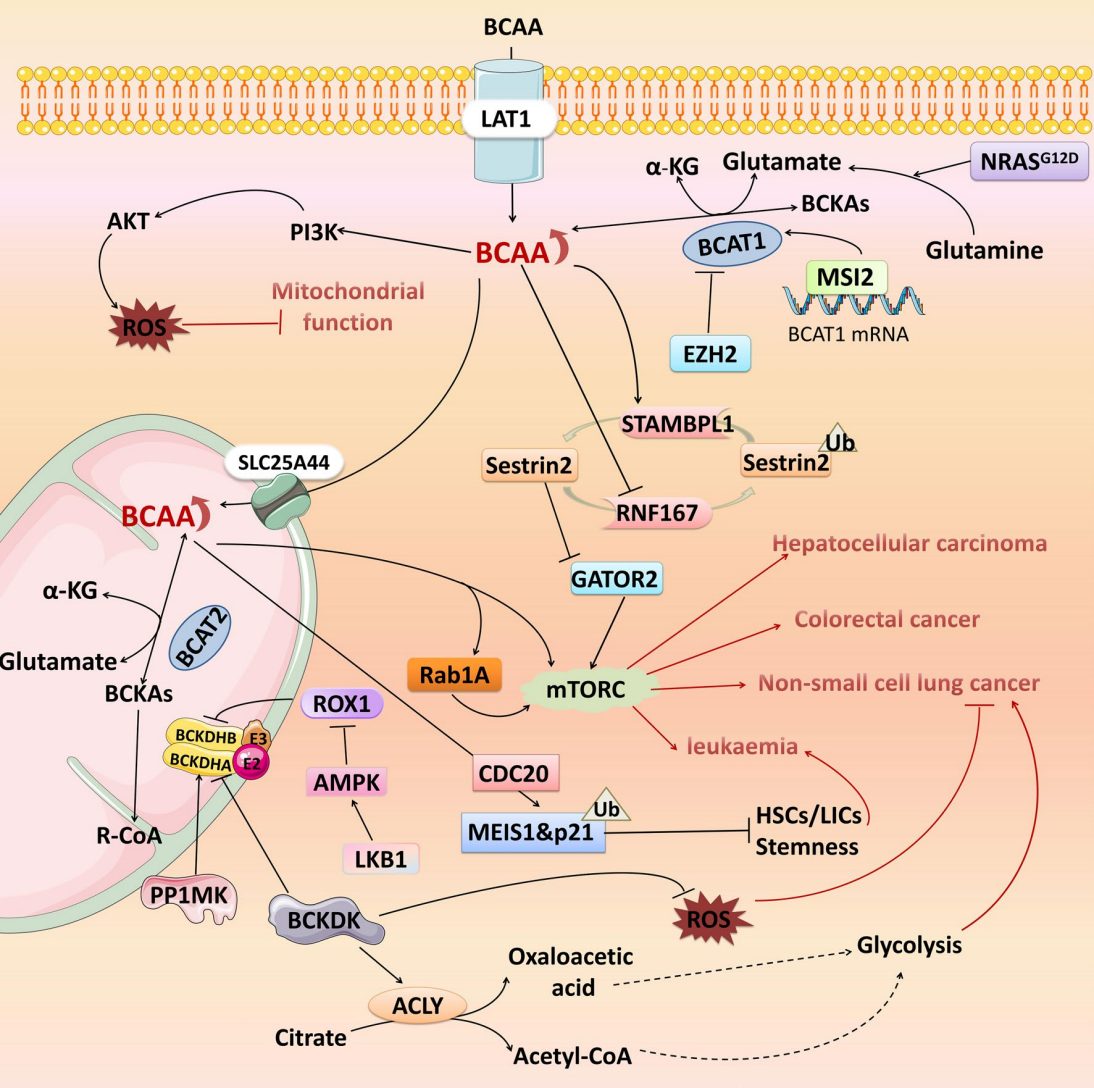
BCAA hypocatabolism promotes tumour development. Leucine affects mTORC activity by regulating the ubiquitination state of Sestrin2. BCAA accumulation promotes the progression of hepatocellular carcinoma, colorectal cancer, non-small cell lung cancer, and leukaemia by activating the mTORC signalling pathway. EZH2, MSI2 and NARS^G12D^ are involved in regulating the expression of BCAT1, which leads to an increase in the catabolic reverse reaction of BCAAs and then promotes the activation of mTORC. The downregulation of BCAT2, BCKDHA and BCKDHB led to a reduction in BCAA catabolism and promotes the activation of mTORC. Deletion of PPM1K leads to BCAA accumulation by increasing the ubiquitination of MEIS1 and p21, damaging the dry nature of HSCs and LICs and leading to leukaemia. In addition, BCAA accumulation promotes ROS production through the PI3K/AKT signalling pathway, leading to mitochondrial dysfunction. BCAA accumulation caused by the overexpression of BCKDK may protect NSCLC cells by maintaining glycolysis and reducing ROS accumulation

The first step in BCAA catabolism is an increase in BCKAs reverse response or a decrease in BCAA catabolism, resulting in increased BCAA levels in plasma and tumour tissue, which is closely related to tumour formation and progression. Specifically, higher circulating levels of total BCAAs, isoleucine, leucine, and valine have a significant causal relationship with the risk of developing squamous cell lung cancer [69]. Plasma BCAA concentrations are significantly elevated in hepatocellular carcinoma (HCC) patients and have been identified as biomarkers for this disease [70]. Elevated BCAA levels are associated with an increased risk of mortality in CRC patients and more than double the risk of developing pancreatic cancer [42, 71]. Furthermore, in pancreatic ductal adenocarcinoma (PDAC) cells, BCAAs can be used as a carbon source to induce lipid synthesis, fulfilling the need for the rapid biomembrane synthesis required for tumour cell proliferation [72]. Elevated levels of BCAAs have been observed in the plasma of breast cancer patients. Compared with those in adjacent normal tissues, the level of BCAAs and the expression level of BCAT1 in cancer tissues are also greater [73], suggesting that the increase in BCAAs levels may be due to the readjustment of the expression and activity of metabolic enzymes involved in the BCAA metabolic pathway (Fig. 2).

#### Activation of the mTORC1 signalling pathway

mTORC1 and AMP-activated protein kinase (AMPK) signalling pathways are major intracellular energy sensing mechanisms. The activation of mTORC1 requires the stimulation of nutrients and growth factors to promote cell growth and regulate the synthesis of proteins, nucleotides and lipids, as well as the processes of autophagy and angiogenesis [74, 75]. Moreover, the mTORC pathway serves as a crucial regulator of cellular immune function and is involved in the activation, differentiation and metabolism of T cells [76, 77]. AMPK is activated under nutrient deficiency conditions and responds to energy stress by inhibiting cell growth and biosynthesis processes. In part, it inhibits the mTORC signalling pathway by promoting BCAA catabolism [78, 79]. Therefore, when exploring the impact of BCAA metabolic reprogramming on cancer, we cannot overlook the pivotal role of the mTORC1 signalling pathway in its regulation. Recent studies revealed that leucine affects the activity of mTORC in colorectal cancer (CRC) by regulating the ubiquitination status of Sestrin2. When leucine is scarce, the E3 ubiquitin ligase RING finger protein 167 (RNF167) catalyses Sestrin2 ubiquitination, promotes Sestrin2 interaction with GATOR2, and inhibits mTORC1 signalling. When leucine is sufficient, STAM-binding-protein-like 1 (STAMBPL) removes the ubiquitin chain on Sestrin2 and activates the mTORC1 signal [80]. In addition, when leucine uptake is reduced or energy production is reduced due to catabolism obstruction, mTORC activity is inhibited by regulating the AMPK signalling pathway [81]. Studies have shown that AMPK directly phosphorylates Raptor to inhibit its activity, which is necessary for energy stress-induced mTORC1 inhibition and tumorigenesis [79].

Importantly the accumulation of BCAAs may be due to increased expression of BCATs, and elevated levels of the substrates BCKAs and glutamate promote the reverse reaction of BCAA metabolism. Studies have shown that zeste homolog 2 (EZH2), an enzymatic subunit of the polycomb repressive complex 2 (PRC2), can epigenetically silence BCAT1 during normal haematopoiesis [66]. Activation of the NRAS gene G12D mutation promotes the conversion of glutamine to glutamate [66]. EZH2 deficiency leads to BCAT1 reactivation in combination with NRAS^G12D^ to promote the BCAT1-catalysed conversion of BCKAs to BCAAs. This maintains high intracellular levels of BCAAs, thereby promoting mTORC1 signalling and inducing the transformation of myeloproliferative neoplasms into leukaemia [66]. Additionally, another study reported that the ability to catabolize BCAAs to BCKAs was also reduced in chronic myeloid leukaemia (CML) patients. The oncogenic RNA-binding protein Musashi2 (MSI2) promotes the reamination of BCKAs to BCAAs by upregulating BCAT1 expression at the translational level, thereby activating the mTORC1 signalling pathway and driving the malignant progression of CML [67].

In addition, the accumulation of BCAAs may also result from decreased expression of enzymes involved in BCAA catabolism, leading to attenuated BCAA catabolism. Compared with those in normal tissues, BCAT2 mRNA and protein levels in tumour tissues of colorectal cancer patients were reduced. Downregulation of BCAT2 expression leads to the accumulation of BCAAs, activation of the mTORC1 signalling pathway, and promotion of CRC tumorigenesis [27]. Furthermore, downregulation of BCKDH complex expression in hepatocellular carcinoma (HCC) tumour tissues leads to the accumulation of BCAAs, thereby activating the mTORC1 signalling pathway and promoting tumour cell growth [65]. Another study revealed that the downregulation of BCKDHA expression attenuates BCAA catabolism and promotes tumour proliferation by promoting the Rab1A-mTORC1 signalling pathway in non-small cell lung cancer (NSCLC) [82]. Among the key factors in this process, Rab1A is a small GTPase that regulates the mTORC1 signalling pathway in cells through amino acid signalling [83]. Defects in the LKB1-AMPK axis activate Prospero-related homeobox 1 (PROX1), inhibit the expression of BCAA catabolic genes such as BCKDHB, and maintain the BCAA pool to activate mTORC, leading to the development and invasion of HCC [84].

#### Epigenetic remodelling

Abnormal epigenetic regulation may result in the modulation of cellular metabolic pathways, thereby affecting intracellular energy production and utilization, as well as signalling pathway selection [85]. However, reduced BCAA catabolism can also induce epigenetic remodelling and promote tumour progression. The elevation of BCAA levels caused by PPM1K deficiency-mediated dysfunction of BCKDH upregulates E3 ubiquitin ligase member cell division cycle 20 (CDC20), which in turn mediates the ubiquitination of MEIS1 and p21. This process suppresses glycolysis and quiescence in haematopoietic stem cells (HSCs) and leukaemia-initiating cells (LICs), thereby impairing their stemness and inhibiting the development of leukaemia [31].

#### Regulation of oxidative stress

Elevated levels of BCAAs can modulate intracellular oxidative stress. Research has shown that increased levels of BCAAs in peripheral blood mononuclear cells promote the production of mitochondrial reactive oxygen species (ROS) by activating the PI3K/Akt signalling pathway, leading to mitochondrial dysfunction [86]. However, the overexpression of BCKDK not only leads to the accumulation of BCAAs, but also enables citric acid to generate acetyl-CoA and oxaloacetic acid by regulating ATP-citrate lyase (ACLY) activity. Together, these two aspects maintain glycolysis, reduce ROS accumulation, and protect NSCLC cells from apoptosis [10, 87].

### BCAA hypercatabolism promotes tumour development

**Fig. 3:**
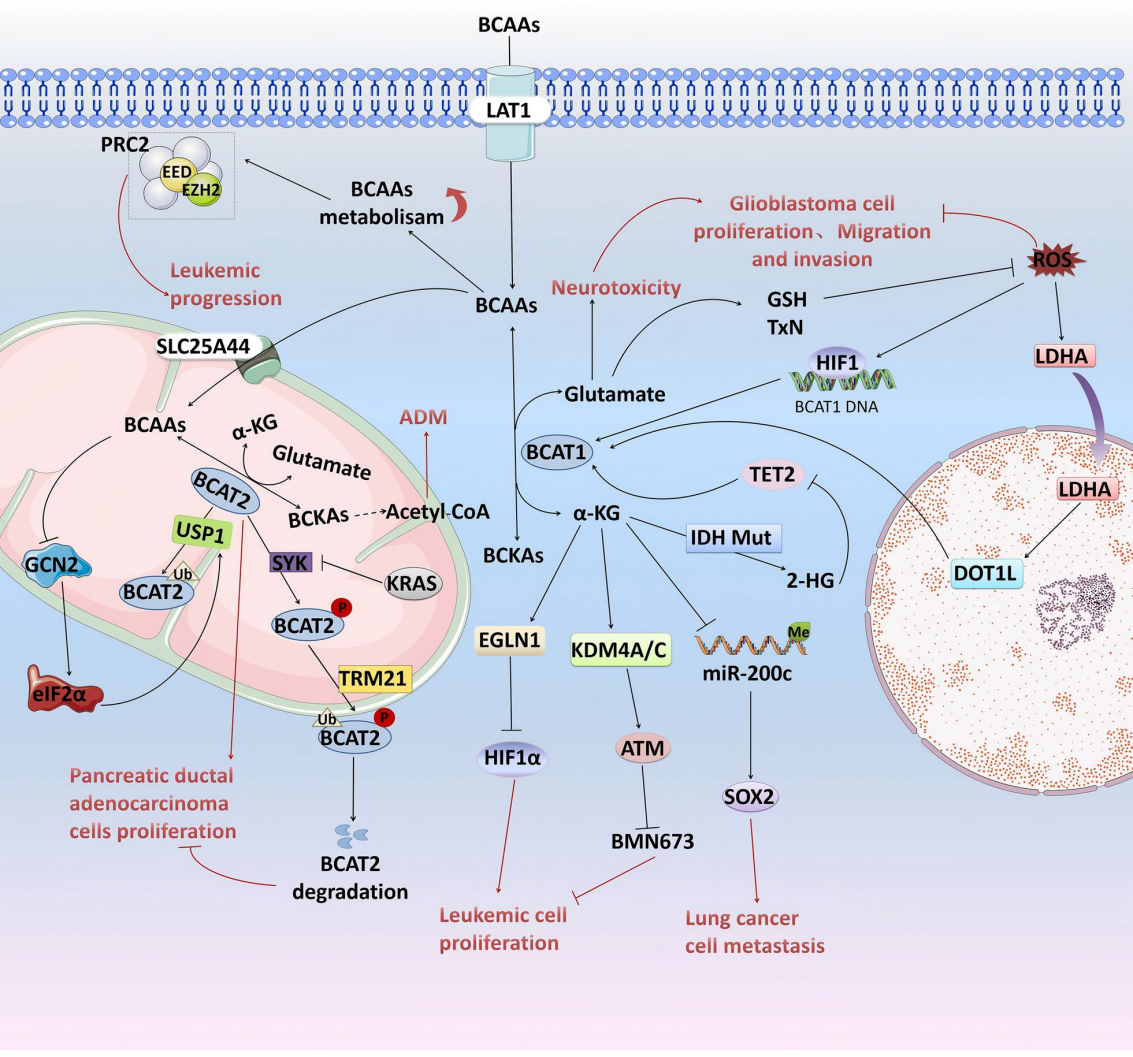
BCAA hypercatabolism promotes tumour development. BCAA metabolism maintains PRC2 activity by promoting the transcription of EZH2 and EED, thereby maintaining the dryness of acute leukaemia. High expression levels of BCAT1 lead to a decrease in α-KG levels, and promote the occurrence and development of leukaemia and lung cancer through epigenetic remodelling of the EGLN1-HIF1α axis, KDM4A/C-ATM axis and miR-200c-SOX2 axis. The BCAA-GCN2-EIF2α axis and the KRAS-SYK axis promote the development of pancreatic ductal adenocarcinoma by stabilizing BCAT2 and enhancing BCAA catabolism. On the one hand, ROS promotes the transcription of BCAT1 by activating HIF1; on the other hand, it up-regulates the expression of BCAT1 through the LDHA-DOT1L axis to increase the catabolism of BCAAs, resulting in an increase in the levels of the glutamate-derived antioxidants GSH and TxN, and maintaining the redox state of glioblastoma

In contrast to the low BCAA catabolism-mediated promotion of tumour progression described above, some tumour cells exhibit increased BCAA catabolism to meet their energy and growth demands. For example, under glutamine deprivation conditions, increased BCAA catabolism aids the survival of liver cancer cells [17]. Additionally, increased BCAA catabolism leads to a decrease in the intermediate metabolite α-KG and an increase in glutamate. Changes in intermediate metabolites not only impact cellular energy metabolism but also promote tumorigenesis at the molecular level by participating in epigenetic regulation and modulating oxidative stress (Fig. 3)**.**

#### Epigenetic remodelling

BCATs not only control the relative expression levels of BCAAs and BCKAs but also regulate the balance between intracellular α-ketoglutarate (α-KG) levels. α-KG not only serves as an intermediate metabolite in the tricarboxylic acid cycle but also acts as a rate-limiting substrate for the α-KG-dependent dioxygenase family. These enzymes play crucial roles in hypoxic signal transduction, maintaining cellular redox homeostasis, and epigenetic modifications [88]. Dysregulation of α-KG levels leading to histone and DNA methylation has been demonstrated to facilitate malignant progression in glioblastoma and acute myeloid leukaemia [33, 36]. The overexpression of BCAT1 depletes α-KG and is closely associated with the upregulation of sex-determining region Y-box 2 (SOX2) [16]. SOX2, an important transcription factor for maintaining cancer stem cell (CSC) plasticity, plays a crucial role in regulating stemness and promoting the metastasis of lung cancer and glioblastoma cells [89]. BCAT1-mediated reduction in α-KG levels can increase promoter methylation of the miR-200c gene, thereby promoting SOX2 expression and leading to the metastasis of lung cancer cells [16]. Other studies have shown that reducing α-KG levels can hinder the degradation of HIF1α via α-KG-dependent dioxygenases such as the Egl-9 family of hypoxia-inducing factor 1 (EGLN1), thus maintaining leukaemia cell growth and survival [33]. Furthermore, in isocitrate dehydrogenase (IDH)-mutant glioblastomas (GBMs), BCAT1 expression is significantly suppressed, possibly because of the pattern of α-KG-mediated BCAT1 methylation [35]. IDH mutations reduce α-KG to the oncometabolite 2-hydroxyglutaric acid (2-HG) [90]. Since α-KG is a necessary cofactor for TET DNA demethylases [36, 91], the competitive inhibition of these enzymes by 2-HG results in high methylation of the BCAT1 promoter in IDH-mutant GBM [92]. These expression trends are similar to those observed in IDH-mutant acute myeloid leukaemia [93]. In contrast, in GBM cells with wild-type (WT) IDH, BCAT1 overexpression promotes tumour cell proliferation by enhancing BCAA metabolism and increases neurotoxic damage to peripheral neurons by promoting glutamate efflux [35]. Moreover, recent studies have revealed that in AML, high expression levels of BCAT1 reduce intracellular α-KG levels and increase H3K9me3 levels by decreasing KDM4A/KDM4C histone lysine demethylase activity, thus suppressing the function of the important DNA damage repair molecule ataxia-telangiectasia mutated protein (ATM) and increasing the sensitivity of cells to the polyadenosine diphosphate ribose polymerase (PARP) inhibitor BMN673 [94].

In addition, research has shown that PDAC cells are highly dependent on the BCAA catabolic pathway. In pancreatic acinar cells driven by KRAS gene mutations, increased levels of histone acetylation leading to acinar-to-ductal metaplasia (ADM) are associated with elevated levels of acetyl-CoA, with leucine being the primary source of acetyl-CoA [95]. BCAT2 is upregulated in pancreatic intraepithelial neoplasia ductal cells [41, 68], subsequently affecting the concentration of acetyl-CoA derived from BCAAs [68]. Another study reported that KRAS suppresses the phosphorylation of BCAT2 induced by spleen tyrosine kinase (SYK), reducing the binding of BCAT2 to the E3 ligase tripartite motif-containing protein 21(TRIM21). This results in decreased ubiquitination of BCAT2, thereby maintaining BCAA catabolism and mitochondrial respiration, which in turn promotes the malignant transformation of PDAC cells [68]. High concentrations of BCAA increase the translation of ubiquitin-specific peptidase 1 (USP1) by inhibiting the GCN2-eIF2α signalling pathway, and USP1 in turn deubiquitinates BCAT2 at the K229 site to stabilize its protein expression, leading to increased catabolism of BCAAs to promote PDAC cell proliferation [41]. Additionally, BCAA metabolism promotes the transcription of the PRC2 components EZH2 and embryonic ectoderm development (EED), maintaining the activity of the general epigenetic regulator PRC2, which is crucial for the maintenance of stem cell characteristics. PRC2 maintains the expression of stem cell-related genes in human AML and acute lymphoblastic leukaemia (ALL) cells to promote tumour progression by catalysing H3K27me3 of target genes [28, 96].

#### Regulation of oxidative stress

The accumulation of ROS is usually detrimental to tumour cell growth; therefore, tumour cells need to upregulate the expression of various antioxidants to maintain redox homeostasis. Glutamate is a major source of the antioxidants GSH and thioredoxin (TxN) [97]. It has been shown that ROS-activated hypoxia-inducible factor-1 (HIF-1) can bind to the hypoxia response element (HRE) within the first intron of the BCAT1 gene and promote its transcription [98]. BCAT1 upregulation-mediated reprogramming of BCAA metabolism is a critical mechanism for maintaining glutamate levels in GBM cells, supporting reductive metabolism and subsequently facilitating tumour cell proliferation, migration, and invasion [37]. Additionally, recent studies have reported that ROS accumulation leads to the translocation of lactate dehydrogenase A (LDHA) to the nucleus, thereby promoting histone H3K79 hypermethylation induced by the methyltransferase disruptor of telomeric silencing-1-like (DOT1L) [99]. Previous research has identified BCAT1 as a downstream effector molecule of DOT1L that mediates the stem cell-like characteristics and migratory capacity of breast cancer cells [21]. In GBM, DOT1L upregulates BCAT1 expression and enhances BCAA catabolism, leading to an increase in TxN indirectly derived from glutamate to balance the redox state of GBM and promote tumour cell proliferation [99].

## BCAA metabolic reprogramming and tumour drug resistance and immunity

### BCAA metabolism and drug resistance in tumours

Tumour drug resistance is a major obstacle to the effective treatment of cancer. Chemotherapeutic drugs usually work by inhibiting the proliferation of tumour cells, killing them or blocking their growth. However, most tumours initially respond to chemotherapy but eventually develop resistance to treatment. Studies have shown that the chemoresistance of certain tumour cells is associated with the reprogramming of BCAA metabolism (Table 1).

**Table 1:** BCAA metabolism and drug resistance in tumours

Chemotherapeutic	Tumour type	BCAA-metabolizing enzyme levels	Chemo-resistance mechanism	References
Cisplatin	Hepatocellular carcinoma	BCAT1 upregulation	Inhibition of mTORC signalling pathway to activate autophagy	[19]
Carboplatin	Epithelial ovarian cancer	BCAT1 upregulation	Upregulation of AKR1C1 expression and reduced accumulation of ROS	[9, 104]
TKI treatment	Lung cancer	BCAT1 upregulation	Reduced accumulation of ROS	[14]
Paclitaxel	Epithelial ovarian cancer and breast cancer	BCKDK upregulation	Enhancement of the mTORC1-Aurora pathway to promote mitosis in tumour cells	[46]
Tamoxifen	Breast cancer	SLC7A5 upregulation	Increased leucine uptake mediates the adaptation of ERα^+^ breast cancer to nutritional stress	[20]

Studies have shown that the estrogen-E2-induced scaffold protein LLGL2 promotes SLC7A5 expression and increases leucine uptake by forming trimers with the leucine transporter SLC7A5 and the NSF attachment protein receptor family member YKT6 in the cytoplasm. This activity mediates the adaptation of ERα^+^ breast cancer to nutritional stress and resistance to tamoxifen treatment [20]. KMH-233 is a selective inhibitor of LAT1 that effectively reduces the protein levels of mTOR in cancer cells. It can enhance the antiproliferative effects of cisplatin and phenylalanine in breast cancer cells by exerting their combined effects [100, 101]. BCAT1 was found to be transcriptionally upregulated in HCC in response to the overexpression of the transcription factor c-Myc, which has been shown to promote the proliferation of liver cancer cells and reduce their sensitivity to cisplatin [64]. Mechanistically, cisplatin induces the upregulation of BCAT1 expression, leading to increased BCAA catabolism. This inhibits mTORC signalling, thereby activating autophagy and countering cisplatin-induced cell death [19]. Furthermore, studies have reported that BCAT1 upregulation is associated with adverse therapeutic responses to treatment with sublethal tyrosine kinase inhibitors (TKIs) in lung cancer cells with epidermal growth factor receptor (EGFR) mutations [14]. Mechanistically, the negative regulation of BCAT1 by H3K9 methylation is weakened, leading to increased glutamate synthesis. Concurrently, it enhances the synthesis of the crucial intracellular antioxidant GSH from glutamate via the glutamate-cysteine ligase catalytic subunit (GCLC), thereby eliminating ROS and mediating TKI resistance [14]. In addition, in epithelial ovarian cancer (EOC), knockdown of BCAT1 can significantly suppress the expression of aldo–keto reductase family 1 member C1 (AKR1C1) [9]. Previous studies have clearly demonstrated that AKR1C1 induces carboplatin resistance in human EOC cells by reducing ROS accumulation [102]. In addition, BCKDK expression is upregulated in chemotherapy-resistant ovarian cancer and decreases the sensitivity of breast and ovarian cancer cells to paclitaxel [45, 46]. Inhibition of BCKDK or a reduction in BCAA levels can synergize with paclitaxel to impede tumour cell mitosis by inhibiting the mTORC1-Aurora pathway [46]. Aurora is a mitotic kinase that functions in the assembly of spindles and centrosomes, as well as in the establishment of a spindle assembly checkpoint, ensuring proper operation of the spindle apparatus during mitosis and accurate segregation of chromosomes [103].Therefore, targeted therapy involvig BCAA uptake and metabolism-related proteins may increase tumour sensitivity to chemotherapy drugs.

### BCAA metabolism and tumour immunity

BCAAs are essential nutrients required by immune cells during organ development, tissue homeostasis and the immune response. In the tumour microenvironment, metabolic reprogramming profoundly affects the function of immune cells, and BCAA metabolic reprogramming is a significant potential mechanism leading to impaired antitumour immune function [13]. The regulatory mechanisms of BCAA metabolic reprogramming and the effects of this reprogramming on the phenotype and function of tumour immune cells differ among various immune cell subsets (Table 2).

**Table 2:** BCAA metabolism and tumour immunity

Immune cell	BCAA-metabolizing enzyme levels	Metabolite levels	Biological function	References
CD4^+^ T cell	BCAT1 upregulation	Decreased BCAA	Inhibit T-cell differentiation	[76]
Foxp3^+^ Treg cell	Slc3A2 upregulation	Decreased BCAA	Maintain the proliferation and function of Treg cells	[106]
CD8^+^ T cell	N/A	Decreased BCAA	Mediate IFNγ-induced antitumour immunity	[15]
	BCAT1 upregulation	N/A	Inhibit CD8^+^ T infiltration and participate in immunosuppression	[108]
	BCAT2 upregulation	N/A	Down-regulating the expression of chemokines associated with CD8 + T cells, reducing the chemotactic ability of CD8^+^ T cells, and weakening the cytotoxic ability of CTL	[8]
B cell	Slc7A5 upregulation	Decreased leucine	Activation of mTORC1 promotes B- cell differentiation and supports IgG and cytokine production	[109]
LARS B cell	N/A	Decreased leucine	Promoting the expression of TGF-β1, leading to immunosuppressive TME and tumour escape	[12]
Macrophages	BCAT1 downregulation	N/A	Downregulated the expression of immune-response gene IRG1 and inhibited the proinflammatory function	[110, 111]
	N/A	Decreased BCKA	Attenuated the phagocytosis activity of macrophages	[39]

#### T lymphocytes

Leucine-mediated activation of mTORC is a key regulator of T-cell activation, differentiation and metabolism [105]. TCR signalling upregulates the expression of BCAT1 in CD4^+^ T cells, leading to increased BCAA transamination, which suppresses the mTORC signalling pathway and glycolysis rate, thereby inhibiting T-cell differentiation [76]. Additionally, Slc3A2, which interacts with LAT1 to form a neutral amino acid transporter, is highly expressed in Foxp3^+^ regulatory T (Treg) cells. It promotes the transport of BCAAs into Tregs, targeting the mTORC signalling pathway to maintain Treg cell proliferation and function [106]. BCAAs upregulate glucose transporter 1 (Glu1) and increase glucose uptake by activating the PI3K/AKT/FOXO1 axis and the mTORC signalling pathway [11]. The expression of IFNγ and its differentiation into the effector state in CD8^+^ T cells are glucose dependent [107], the accumulation of BCAAs in CD8^+^ T cells enhances antitumour immunity in lung cancer xenograft mouse models by increasing glycolysis and oxidative phosphorylation [11]. BCAT1 is a downstream regulatory factor of interferon-gamma (IFNγ)-mediated CSC plasticity in breast cancer. IFNγ released by activated T cells not only promotes the transformation of non-CSCs to CSCs but also enhances the sphere formation capacity, resistance to radiotherapy and chemotherapy, and the metastatic ability of CSCs. The combination of cancer immunotherapy with BCAT1 inhibitors can induce antitumour T-cell responses while blocking IFNγ-induced immune escape [15]. Moreover, studies have reported that the accumulation of BCAAs increases the effector function of CD8^+^ T cells and promotes antitumour immunity by reprogramming glucose metabolism. Furthermore, knocking out BCAT1 may inhibit tumour growth by promoting the differentiation of glioblastoma cells and sustaining the continuous infiltration of CD8^+^ T cells in the immune microenvironment [108]. Research indicates that BCAT2 can reduce the chemotactic ability of CD8^+^ T cells and weaken the cytotoxicity of cytotoxic T lymphocytes (CTLs) by downregulating the expression of CD8^+^ T-cell-associated chemokines, such as CCL3, CCL4, CCL5, CXCL9, and CXCL10. This process may contribute to the progression of bladder cancer. The combination of BCAT2 deletion and an anti-PD-1 antibody treatment effectively blocks tumour growth in vivo [8]. This finding suggested that the inhibition of BCAT2 may enhance immunotherapeutic effects.

#### B lymphocytes

Leucine is transported into B cells via SLC7A5, activating mTORC1 to promote B-cell differentiation and support the production of IgG and cytokines [109]. Additionally, leucine can induce the generation of a B-cell subset that highly expresses leucine-tRNA-synthetase-2 (LARS2), promoting CRC immune evasion, tumour growth and progression in a TGF-β1-dependent manner [12]. Mechanistically, leucine increases the activity of the key transcription factor PAX5 in B-cell development and differentiation by promoting LARS2-dependent mitochondrial NAD + regeneration and upregulating the expression of the deacetylase sirtuin-1 (SIRT1), thereby promoting TGF-β1 production [12]. This leads to the formation of an immunosuppressive tumour microenvironment and tumour immune evasion.

#### Macrophages

During lipopolysaccharide-induced macrophage activation, BCAT1 inactivation can induce ROS production, which subsequently downregulates the expression of the immune-responsive gene 1 protein (IRG1) and inhibits the proinflammatory functions of macrophages [110, 111]. Furthermore, in GBM cells with high BCAT1 expression, BCKAs are excreted from cells via monocarboxylate transporter 1 (MCT1) and are subsequently taken up by tumour-associated macrophages and reamidated from BCAAs. BCKAs attenuate the phagocytic activity of macrophages, thereby promoting GBM growth [39]. Therefore, BCAA metabolism is crucial for maintaining the function of immune cells.

## Clinical and in vivo studies of BCAA supplement therapy and metabolic enzyme inhibitors

At present, many clinical studies have reported that appropriate supplementation of BCAAs is beneficial for improving the prognosis of patients with liver cancer [112]. Oral supplementation with BCAA granules can reduce the risk of developing liver cancer in patients with cirrhosis [113]. Oral BCAA supplementation protects liver function and reduces the risk of relapse and complications after radiofrequency ablation of HCC [114]. In addition, supplementation with BCAA may serve as a useful adjunctive therapy to improve the prognosis of patients with advanced HCC treated with sorafenib [115]. These methods appear appear to be useful for improving the prognosis of patients with HCC because they can be applied safely without significant side effects.

At present, the application of branched-chain amino acid inhibitors in tumour therapy is still in the preclinical research stage. However, studies have demonstrated its potential therapeutic effects through in vivo experiments, laying the foundation for future clinical applications. A recent study reported that the small-molecule drug Eupalinolide B (EB) can act as an inhibitor that targets BCAT1 to induce the apoptosis of triple-negative breast cancer (TNBC) cells both in vivo and in vitro [116]. In addition, WQQ-345, a novel BCAT1 inhibitor, has antitumour activity in vitro and in vivo against TKI-resistant lung cancer with high levels of BCAT1 expression [117]. The BCAT2 inhibitor 2-Aryl Benzimidazole improves the progression of PDAC in mice [118, 119] (Table 3).

**Table 3:** Clinical and in vivo studies of BCAA supplement therapy and metabolic enzyme inhibitors

Therapy type	Drug name	Target	Efficacy	Research type	References
Supplemention	BCAA supplemention	N/A	Reduce the risk of liver cancer in patients with cirrhosis	Clinical study	[112]
			Reduce the risk of recurrence and complications in patients with HCC after radiofrequency ablation		[113]
			Improve the prognosis of patients with advanced HCC		[114]
Inhibitor	JPH203	LAT1	Inhibit the proliferation of advanced solid tumours and improve prognosis	Clinical study	[56, 57]
	Eupalinolide B	BCAT1	Induced TNBC cell apoptosis	In vivo/in vitro experiments (mouse model)	[116]
	WQQ-345	BCAT1	Antitumor activity		[117]
	2-Aryl Benzimidazole	BCAT2	Improves the progression of PDAC mice		[118, 119]

## Conclusion

Alterations in cellular metabolism can promote cell transformation and tumour progression. Metabolic phenotypes can also be used to provide prognostic information and treat cancer [120]. Therefore, studies on tumour metabolism are important for understanding the pathogenesis of different tumours and for improving clinical treatment. The role of BCAAs is not limited to their contribution as essential amino acids; they also play an important role in the body by providing a source of nitrogen and carbon, supporting the synthesis of other molecules such as amino acids and nucleotides, and maintaining energy balance [59, 121]. In addition, they can also act as signalling molecules and influence tumour progression by affecting the epigenetic landscape, oxidative stress, drug resistance, and immune responses [122–124].

There are significant differences in the degree of BCAA metabolic reprogramming across tumour types. Numerous factors influence BCAA metabolism, including alterations in the expression or activity of BCAA transporters and related metabolic enzymes, transcription factors, oncogenes and tumour suppressor genes; alterations in the tumour phenotype; the alterations in the tumour microenvironment [125]. Moreover, the type of dysregulation of BCAA metabolism appears to differ among various types, as it may involve reduced BCAA catabolism, leading to BCAA accumulation or enhanced BCAA uptake and catabolism [66–68]. This may involve the extent to which tumour cells utilize intracellular or circulating BCAAs. The current paper summarizes the multiple mechanisms by which alterations in BCAA metabolism mediate tumorigenesis and discusses potential therapeutic targets.

We discussed numerous specific tumour-dependent pathways associated with BCAA metabolism, and provided insight into tumour-specific pathogenesis mechanisms. However, the clinical application of effective BCAA metabolism therapies remains limited. One challenge is the significant heterogeneity of BCAA metabolism under different conditions. This requires the comprehensive consideration of factors such as tumour type, tumour microenvironment features, and tumour stage to determine the stage of tumour progression. In addition, whether BCAA metabolic reprogramming synergizes with other mechanisms to promote tumour growth under different circumstances should be studied. Given the importance of BCAA metabolic reprogramming in tumour progression, metabolic enzymes involved in BCAA metabolism may be potential therapeutic targets for the treatment of cancers.

Interestingly, cancer stem cells are a distinct subpopulation within tumours, that can self-renew and differentiae, and they drive tumour migration, therapy resistance, and immune evasion [126]. The CSC niche is a specific tumour microenvironment composed of the extracellular matrix, stromal cells (including cancer-associated fibroblasts and immune cells), cytokines, and growth factors. These components maintain the stemness, self-renewal, and cell fate determination of CSCs through signalling pathways such as the WNT, NOTCH, and BMP pathways [126]. Additionally, nutrients, as essential components of the stem cell niche, influence stem cell fate and function through various mechanisms [127]. Although direct evidence is currently lacking that BCAA metabolic reprogramming directly regulates the aforementioned signalling pathways involved in modulating cancer stem cells, BCAAs and their metabolic enzymes can reshape the CSC ecological niche of tumour cells by regulating the expression of CSC markers such as SOX2, DOT1L, and PRC2, as well as modulating immune cell function to impact the construction of the CSC niche. Suppression of CSC ecological niche by altering BCAA metabolism may be an important strategy to prevent tumour recurrence and metastasis.

Additionally, immunotherapy, as an emerging and promising cancer treatment, has attracted widespread attention. In the future, more in-depth research will further unveil a broader understanding of the profound impacts of BCAA metabolic reprogramming on tumour immunity and uncover new methods to enhance tumour immunotherapy. This may involve identifying novel therapeutic targets, developing more effective treatment strategies, and exploring the potential for personalized therapy, thereby promoting the start of a new era in cancer treatment.

## Data Availability

Not applicable.

## Abbreviations

BCAA: Branched chain amino acid
Ieu: Leucine
Ile: Isoleucine
LAT: L-type amino acid transporter
HCC: Hepatocellular carcinoma
BC: Breast Cancer
GPRC5C: Gprotein coupled receptor family C group 5 member C
AML: Acute myeloid leukaemia
BCATs: Branched-chain aminotransferases
α-KG: α-Ketoglutaric acid
BCAT1: Branched chain aminotransferase 1
BCAT2: Branched chain aminotransferase 2
BCKDH: Branched chain alpha-ketoate dehydrogenase
PPM1K: Mitochondrialtargeted protein phosphatase Mg2+ and Mn2+ - dependent 1K
mTOR: Mammalian target of rapamycin
RNF167: RING finger protein 167
STAMBPL1: STAM-binding-protein-like 1
CRC: Colorectal cancer
PDAC: Pancreatic ductal adenocarcinoma
EZH2: Zeste homolog 1
PRC2: Polycomb Repressive Complex 2
CML: Chronic myeloid leukemia
MSI2: Musashi 2
NSCLC: Non-small cell lung cancer
HSCs: Hematopoietic stem cells
LICs: Leukaemia-initiating cells
ROS: Reactive oxygen species
ACLY: ATP-citrate lyase
SOX2: Sex determining region Y-box 2
EGLN1: Egl-9 family of hypoxia-inducing factor1
IDH: Isocitrate dehydrogenase
Mut: Mutant
GBM: Glioblastom
2-HG: 2-Hydroxyglutaric acid
WT: Wildtype
ATM: Ataxia-telangiectasia mutated proteins
PARP: Poly adenosine diphosphate ribose polymerase
ADM: Acinar-to-Ductal Metaplastic
SYK: Spleen tyrosine kinaseSYK
USP1: Ubiquitin specific peptidase 1
EED: Embryonic ectoderm development
ALL: Acute lymphoblastic leukemia
TxN: Thioredoxin
HIF-1: Hypoxia-inducible factor-1
LDHA: Lactate dehydrogenase A
DOT1L: Disruptor of telomeric silencing-1-like
OS: Overall Survival
FLOT2: Flotillin 2
RBM47: RNA binding motif protein 47
EGFR: Epidermal growth factor receptor
TKI: Tyrosine kinase inhibitors
GCLC: Glutamate-cysteine ligase catalytic subunit
EOC: Epithelial ovarian cancer
AKR1C1: Aldo–keto reductase family 1 member C1
ERα^+^: Estrogen receptor positive
TCR: T cell receptor
Treg: Regulatory T
IFNγ: Interferon gamma
CSC: Cancer stem cell
Glu1: Glucose transporter
CTLs: Cytotoxic T lymphocytes
LARS2: Leucine-t RNA-synthetase-2
SIRT1: Sirtuin - 1
IRG1: Immune-responsive gene 1 protein
MCT1: Monocarboxylate transporters1
YRDC: YrdC N(6)-threonylcarbamoyl transferase domain containing
AMPK: AMP-activated protein kinase
TNBC: Triple negative breast cancer
EB: Eupalinolide B
PROX1: Prospero-related homeobox 1
TRIM21: Tripartite motif-containing protein 21

## References

[1] Pavlova NN, Thompson CB. The emerging hallmarks of cancer metabolism. Cell Metab. 2016;23(1):27–47. 26771115 10.1016/j.cmet.2015.12.006 PMC4715268

[2] Faubert B, Solmonson A, DeBerardinis RJ. Metabolic reprogramming and cancer progression. Science (New York, NY). 2020;368(6487):eaaw5473. 32273439 10.1126/science.aaw5473 PMC7227780

[3] Hanahan D, Weinberg RA. Hallmarks of cancer: the next generation. Cell. 2011;144(5):646–74. 21376230 10.1016/j.cell.2011.02.013

[4] Vazquez A, Kamphorst JJ, Markert EK, et al. Cancer metabolism at a glance. J Cell Sci. 2016;129(18):3367–73. 27635066 10.1242/jcs.181016 PMC6518336

[5] DeBerardinis RJ, Chandel NS. Fundamentals of cancer metabolism. Sci Adv. 2016;2(5):e1600200. 27386546 10.1126/sciadv.1600200 PMC4928883

[6] Sheen J-H, Zoncu R, Kim D, et al. Defective regulation of autophagy upon leucine deprivation reveals a targetable liability of human melanoma cells in vitro and in vivo. Cancer Cell. 2011;19(5):613–28. 21575862 10.1016/j.ccr.2011.03.012 PMC3115736

[7] Taya Y, Ota Y, Wilkinson AC, et al. Depleting dietary valine permits nonmyeloablative mouse hematopoietic stem cell transplantation. Science (New York, NY). 2016;354(6316):1152–5. 27934766 10.1126/science.aag3145

[8] Cai Z, Chen J, Yu Z, et al. BCAT2 shapes a noninflamed tumor microenvironment and induces resistance to anti-PD-1/PD-L1 immunotherapy by negatively regulating proinflammatory chemokines and anticancer immunity. Adv Sci. 2023;10(8):2207155. 10.1002/advs.202207155 PMC10015882 36642843

[9] Wang Z-Q, Faddaoui A, Bachvarova M, et al. BCAT1 expression associates with ovarian cancer progression: possible implications in altered disease metabolism. Oncotarget. 2015;6(31):31522–43. 26372729 10.18632/oncotarget.5159 PMC4741622

[10] Wang Y, Xiao J, Jiang W, et al. BCKDK alters the metabolism of non-small cell lung cancer. Transl Lung Cancer Res. 2021;10(12):4459–76. 35070754 10.21037/tlcr-21-885 PMC8743533

[11] Yao C-C, Sun R-M, Yang Y, et al. Accumulation of branched-chain amino acids reprograms glucose metabolism in CD8+ T cells with enhanced effector function and anti-tumor response. Cell Rep. 2023;42(3):112186. 36870057 10.1016/j.celrep.2023.112186

[12] Wang Z, Lu Z, Lin S, et al. Leucine-tRNA-synthetase-2-expressing B cells contribute to colorectal cancer immunoevasion. Immunity. 2022;55(6):1067-1081.e8. 35659337 10.1016/j.immuni.2022.04.017

[13] Yang L, Chu Z, Liu M, et al. Amino acid metabolism in immune cells: essential regulators of the effector functions, and promising opportunities to enhance cancer immunotherapy. J Hematol Oncol. 2023;16(1):59. 37277776 10.1186/s13045-023-01453-1 PMC10240810

[14] Wang Y, Zhang J, Ren S, et al. Branched-chain amino acid metabolic reprogramming orchestrates drug resistance to EGFR tyrosine kinase inhibitors. Cell Rep. 2019;28(2):512-525.e6. 31291585 10.1016/j.celrep.2019.06.026

[15] Beziaud L, Young CM, Alonso AM, et al. IFNγ-induced stem-like state of cancer cells as a driver of metastatic progression following immunotherapy. Cell Stem Cell. 2023;30(6):818-831.e6. 37267916 10.1016/j.stem.2023.05.007

[16] Mao L, Chen J, Lu X, et al. Proteomic analysis of lung cancer cells reveals a critical role of BCAT1 in cancer cell metastasis. Theranostics. 2021;11(19):9705–20. 34646394 10.7150/thno.61731 PMC8490523

[17] Yang D, Liu H, Cai Y, et al. Branched-chain amino acid catabolism breaks glutamine addiction to sustain hepatocellular carcinoma progression. Cell Rep. 2022;41(8):111691. 36417878 10.1016/j.celrep.2022.111691

[18] Zhai M, Yang Z, Zhang C, et al. APN-mediated phosphorylation of BCKDK promotes hepatocellular carcinoma metastasis and proliferation via the ERK signaling pathway. Cell Death Dis. 2020;11(5):396. 32457292 10.1038/s41419-020-2610-1 PMC7249043

[19] Luo L, Sun W, Zhu W, et al. BCAT1 decreases the sensitivity of cancer cells to cisplatin by regulating mTOR-mediated autophagy via branched-chain amino acid metabolism. Cell Death Dis. 2021;12(2):169. 33568627 10.1038/s41419-021-03456-7 PMC7876012

[20] Saito Y, Li L, Coyaud E, et al. LLGL2 rescues nutrient stress by promoting leucine uptake in ER+ breast cancer. Nature. 2019;569(7755):275–9. 30996345 10.1038/s41586-019-1126-2

[21] Oktyabri D, Ishimura A, Tange S, et al. DOT1L histone methyltransferase regulates the expression of BCAT1 and is involved in sphere formation and cell migration of breast cancer cell lines. Biochimie. 2016;123:20–31. 26783998 10.1016/j.biochi.2016.01.005

[22] Biswas D, Slade L, Duffley L, et al. Inhibiting BCKDK in triple negative breast cancer suppresses protein translation, impairs mitochondrial function, and potentiates doxorubicin cytotoxicity. Cell Death Discov. 2021;7(1):241. 34526485 10.1038/s41420-021-00602-0 PMC8443725

[23] Passarelli MC, Pinzaru AM, Asgharian H, et al. Leucyl-tRNA synthetase is a tumour suppressor in breast cancer and regulates codon-dependent translation dynamics. Nat Cell Biol. 2022;24(3):307–15. 35288656 10.1038/s41556-022-00856-5 PMC8977047

[24] Xue P, Zeng F, Duan Q, et al. BCKDK of BCAA catabolism cross-talking with the MAPK pathway promotes tumorigenesis of colorectal cancer. EBioMedicine. 2017;20:50–60. 28501528 10.1016/j.ebiom.2017.05.001 PMC5478211

[25] Murata K, Moriyama M. Isoleucine, an essential amino acid, prevents liver metastases of colon cancer by antiangiogenesis. Can Res. 2007;67(7):3263–8. 10.1158/0008-5472.CAN-06-3739 17409434

[26] Tian Q, Yuan P, Quan C, et al. Phosphorylation of BCKDK of BCAA catabolism at Y246 by Src promotes metastasis of colorectal cancer. Oncogene. 2020;39(20):3980–96. 32238881 10.1038/s41388-020-1262-z PMC7220852

[27] Kang Z-R, Jiang S, Han J-X, et al. Deficiency of BCAT2-mediated branched-chain amino acid catabolism promotes colorectal cancer development. Biochim Biophys Acta Mol Basis Dis. 2024;1870(2):166941. 37926361 10.1016/j.bbadis.2023.166941

[28] Kikushige Y, Miyamoto T, Kochi Y, et al. Human acute leukemia uses branched-chain amino acid catabolism to maintain stemness through regulating PRC2 function. Blood Adv. 2023;7(14):3592–603. 36044390 10.1182/bloodadvances.2022008242 PMC10368855

[29] Han L, Dong L, Leung K, et al. METTL16 drives leukemogenesis and leukemia stem cell self-renewal by reprogramming BCAA metabolism. Cell Stem Cell. 2023;30(1):52-68.e13. 36608679 10.1016/j.stem.2022.12.006 PMC9838187

[30] He X, Xu Y, Huang D, et al. P2X1 enhances leukemogenesis through PBX3-BCAT1 pathways. Leukemia. 2023;37(2):265–75. 36418376 10.1038/s41375-022-01759-y PMC9898031

[31] Liu X, Zhang F, Zhang Y, et al. PPM1K regulates hematopoiesis and leukemogenesis through CDC20-mediated ubiquitination of MEIS1 and p21. Cell Rep. 2018;23(5):1461–75. 29719258 10.1016/j.celrep.2018.03.140

[32] Hillier J, Allcott GJ, Guest LA, et al. The BCAT1 CXXC motif provides protection against ROS in acute myeloid leukaemia cells. Antioxidants (Basel, Switzerland). 2022;11(4):683. 35453368 10.3390/antiox11040683 PMC9030579

[33] Raffel S, Falcone M, Kneisel N, et al. BCAT1 restricts αKG levels in AML stem cells leading to IDHmut-like DNA hypermethylation. Nature. 2017;551(7680):384–8. 29144447 10.1038/nature24294

[34] Zhang YW, Velasco-Hernandez T, Mess J, et al. GPRC5C drives branched-chain amino acid metabolism in leukemogenesis. Blood Adv. 2023;7(24):7525–38. 37639313 10.1182/bloodadvances.2023010460 PMC10761356

[35] Tönjes M, Barbus S, Park YJ, et al. BCAT1 promotes cell proliferation through amino acid catabolism in gliomas carrying wild-type IDH1. Nat Med. 2013;19(7):901–8. 23793099 10.1038/nm.3217 PMC4916649

[36] Turcan S, Rohle D, Goenka A, et al. IDH1 mutation is sufficient to establish the glioma hypermethylator phenotype. Nature. 2012;483(7390):479–83. 22343889 10.1038/nature10866 PMC3351699

[37] Zhang B, Chen Y, Shi X, et al. Regulation of branched-chain amino acid metabolism by hypoxia-inducible factor in glioblastoma. Cell Mol Life Sci. 2021;78(1):195–206. 32088728 10.1007/s00018-020-03483-1 PMC8112551

[38] Zhang B, Peng H, Zhou M, et al. Targeting BCAT1 combined with α-ketoglutarate triggers metabolic synthetic lethality in glioblastoma. Can Res. 2022;82(13):2388–402. 10.1158/0008-5472.CAN-21-3868 PMC9256772 35499760

[39] Silva LS, Poschet G, Nonnenmacher Y, et al. Branched-chain ketoacids secreted by glioblastoma cells via MCT1 modulate macrophage phenotype. EMBO Rep. 2017;18(12):2172–85. 29066459 10.15252/embr.201744154 PMC5709768

[40] Lei M-Z, Li X-X, Zhang Y, et al. Acetylation promotes BCAT2 degradation to suppress BCAA catabolism and pancreatic cancer growth. Signal Transduct Target Ther. 2020;5(1):70. 32467562 10.1038/s41392-020-0168-0 PMC7256045

[41] Li J-T, Li K-Y, Su Y, et al. Diet high in branched-chain amino acid promotes PDAC development by USP1-mediated BCAT2 stabilization. Natl Sci Rev. 2022;9(5):nwab212. 35663242 10.1093/nsr/nwab212 PMC9154341

[42] Mayers JR, Wu C, Clish CB, et al. Elevation of circulating branched-chain amino acids is an early event in human pancreatic adenocarcinoma development. Nat Med. 2014;20(10):1193–8. 25261994 10.1038/nm.3686 PMC4191991

[43] Zhu Z, Achreja A, Meurs N, et al. Tumour-reprogrammed stromal BCAT1 fuels branched-chain ketoacid dependency in stromal-rich PDAC tumours. Nat Metab. 2020;2(8):775–92. 32694827 10.1038/s42255-020-0226-5 PMC7438275

[44] Dey P, Baddour J, Muller F, et al. Genomic deletion of malic enzyme 2 confers collateral lethality in pancreatic cancer. Nature. 2017;542(7639):119–23. 28099419 10.1038/nature21052 PMC5398413

[45] Jazaeri AA, Awtrey CS, Chandramouli GVR, et al. Gene expression profiles associated with response to chemotherapy in epithelial ovarian cancers. Clin Cancer Res. 2005;11(17):6300–10. 16144934 10.1158/1078-0432.CCR-04-2682

[46] Ibrahim SL, Abed MN, Mohamed G, et al. Inhibition of branched-chain alpha-keto acid dehydrogenase kinase augments the sensitivity of ovarian and breast cancer cells to paclitaxel. Br J Cancer. 2023;128(5):896–906. 36526674 10.1038/s41416-022-02095-9 PMC9977917

[47] Harper AE, Miller RH, Block KP. Branched-chain amino acid metabolism. Ann Rev Nutr. 1984;4:409–54. 6380539 10.1146/annurev.nu.04.070184.002205

[48] Neinast M, Murashige D, Arany Z. Branched chain amino acids. Annu Rev Physiol. 2019;81:139–64. 30485760 10.1146/annurev-physiol-020518-114455 PMC6536377

[49] Vanweert F, Schrauwen P, Phielix E. Role of branched-chain amino acid metabolism in the pathogenesis of obesity and type 2 diabetes-related metabolic disturbances BCAA metabolism in type 2 diabetes. Nutr Diabetes. 2022;12(1):35. 35931683 10.1038/s41387-022-00213-3 PMC9356071

[50] Zhao X, Zhang X, Pei J, et al. Targeting BCAA metabolism to potentiate metformin’s therapeutic efficacy in the treatment of diabetes in mice. Diabetologia. 2023;66(11):2139–53. 37581618 10.1007/s00125-023-05985-6

[51] Murashige D, Jung JW, Neinast MD, et al. Extra-cardiac BCAA catabolism lowers blood pressure and protects from heart failure. Cell Metab. 2022;34(11):1749-1764.e7. 36223763 10.1016/j.cmet.2022.09.008 PMC9633425

[52] McGarrah RW, White PJ. Branched-chain amino acids in cardiovascular disease. Nat Rev Cardiol. 2023;20(2):77–89. 36064969 10.1038/s41569-022-00760-3 PMC10284296

[53] Kim S-Y, Ong Q, Liao Y, et al. Genetic ablation of LAT1 inhibits growth of liver cancer cells and downregulates mTORC1 signaling. Int J Mol Sci. 2023;24(11):9171. 37298123 10.3390/ijms24119171 PMC10253063

[54] Häfliger P, Charles R-P. The L-type amino acid transporter LAT1—an emerging target in cancer. Int J Mol Sci. 2019;20(10):2428. 31100853 10.3390/ijms20102428 PMC6566973

[55] Kurozumi S, Kaira K, Matsumoto H, et al. Association of L-type amino acid transporter 1 (LAT1) with the immune system and prognosis in invasive breast cancer. Sci Rep. 2022;12(1):2742. 35177712 10.1038/s41598-022-06615-8 PMC8854643

[56] Okano N, Naruge D, Kawai K, et al. First-in-human phase I study of JPH203, an L-type amino acid transporter 1 inhibitor, in patients with advanced solid tumors. Invest New Drugs. 2020;38(5):1495–506. 32198649 10.1007/s10637-020-00924-3

[57] Enomoto K, Sato F, Tamagawa S, et al. A novel therapeutic approach for anaplastic thyroid cancer through inhibition of LAT1. Sci Rep. 2019;9(1):14616. 31601917 10.1038/s41598-019-51144-6 PMC6787004

[58] Adeva-Andany MM, López-Maside L, Donapetry-García C, et al. Enzymes involved in branched-chain amino acid metabolism in humans. Amino Acids. 2017;49(6):1005–28. 28324172 10.1007/s00726-017-2412-7

[59] Rossmeislová L, Gojda J, Smolková K. Pancreatic cancer: branched-chain amino acids as putative key metabolic regulators? Cancer Metastasis Rev. 2021;40(4):1115–39. 34962613 10.1007/s10555-021-10016-0

[60] Shimomura Y, Obayashi M, Murakami T, et al. Regulation of branched-chain amino acid catabolism: nutritional and hormonal regulation of activity and expression of the branched-chain alpha-keto acid dehydrogenase kinase. Curr Opin Clin Nutr Metab Care. 2001;4(5):419–23. 11568504 10.1097/00075197-200109000-00013

[61] Wanders D, Hobson K, Ji X. Methionine restriction and cancer biology. Nutrients. 2020;12(3):684. 32138282 10.3390/nu12030684 PMC7146589

[62] Yan J, Chen D, Ye Z, et al. Molecular mechanisms and therapeutic significance of tryptophan metabolism and signaling in cancer. Mol Cancer. 2024;23(1):241. 39472902 10.1186/s12943-024-02164-y PMC11523861

[63] Wu X, Yuan H, Wu Q, et al. Threonine fuels glioblastoma through YRDC-mediated codon-biased translational reprogramming. Nat Cancer. 2024;5(7):1024–44. 38519786 10.1038/s43018-024-00748-7 PMC11552442

[64] Zheng Y-H, Hu W-J, Chen B-C, et al. BCAT1, a key prognostic predictor of hepatocellular carcinoma, promotes cell proliferation and induces chemoresistance to cisplatin. Liver Int. 2016;36(12):1836–47. 27246112 10.1111/liv.13178

[65] Ericksen RE, Lim SL, McDonnell E, et al. Loss of BCAA catabolism during carcinogenesis enhances mTORC1 activity and promotes tumor development and progression. Cell Metab. 2019;29(5):1151-1165.e6. 30661928 10.1016/j.cmet.2018.12.020 PMC6506390

[66] Gu Z, Liu Y, Cai F, et al. Loss of EZH2 reprograms BCAA Metabolism to drive leukemic transformation. Cancer Discov. 2019;9(9):1228–47. 31189531 10.1158/2159-8290.CD-19-0152 PMC6726547

[67] Hattori A, Tsunoda M, Konuma T, et al. Cancer progression by reprogrammed BCAA metabolism in myeloid leukaemia. Nature. 2017;545(7655):500–4. 28514443 10.1038/nature22314 PMC5554449

[68] Li J-T, Yin M, Wang D, et al. BCAT2-mediated BCAA catabolism is critical for development of pancreatic ductal adenocarcinoma. Nat Cell Biol. 2020;22(2):167–74. 32029896 10.1038/s41556-019-0455-6

[69] Xu H, Wang X, Xu X, et al. Association of plasma branched-chain amino acid with multiple cancers: a Mendelian randomization analysis. Clin Nutr (Edinburgh, Scotland). 2023;42(12):2493–502. 10.1016/j.clnu.2023.10.019 37922693

[70] Nezami Ranjbar MR, Luo Y, Di Poto C, et al. GC-MS based plasma metabolomics for identification of candidate biomarkers for hepatocellular carcinoma in Egyptian cohort. PLoS ONE. 2015;10(6):e0127299. 26030804 10.1371/journal.pone.0127299 PMC4452085

[71] Long L, Yang W, Liu L, et al. Dietary intake of branched-chain amino acids and survival after colorectal cancer diagnosis. Int J Cancer. 2021;148(10):2471–80. 33341092 10.1002/ijc.33449 PMC8213867

[72] Lee JH, Cho Y-R, Kim JH, et al. Branched-chain amino acids sustain pancreatic cancer growth by regulating lipid metabolism. Exp Mol Med. 2019;51(11):1–11. 31784505 10.1038/s12276-019-0350-z PMC6884453

[73] Zhang L, Han J. Branched-chain amino acid transaminase 1 (BCAT1) promotes the growth of breast cancer cells through improving mTOR-mediated mitochondrial biogenesis and function. Biochem Biophys Res Commun. 2017;486(2):224–31. 28235484 10.1016/j.bbrc.2017.02.101

[74] Tian T, Li X, Zhang J. mTOR signaling in cancer and mTOR inhibitors in solid tumor targeting therapy. Int J Mol Sci. 2019;20(3):755. 30754640 10.3390/ijms20030755 PMC6387042

[75] Sivanand S, Vander Heiden MG. Emerging roles for branched-chain amino acid metabolism in cancer. Cancer Cell. 2020;37(2):147–56. 32049045 10.1016/j.ccell.2019.12.011 PMC7082774

[76] Ananieva EA, Patel CH, Drake CH, et al. Cytosolic branched chain aminotransferase (BCATc) regulates mTORC1 signaling and glycolytic metabolism in CD4+ T cells. J Biol Chem. 2014;289(27):18793–804. 24847056 10.1074/jbc.M114.554113 PMC4081922

[77] Ananieva EA, Powell JD, Hutson SM. Leucine metabolism in T cell activation: mTOR signaling and beyond. Adv Nutr (Bethesda, Md). 2016;7(4):798S-805S. 10.3945/an.115.011221 PMC4942864 27422517

[78] González A, Hall MN, Lin S-C, et al. AMPK and TOR: the Yin and Yang of cellular nutrient sensing and growth control. Cell Metab. 2020;31(3):472–92. 32130880 10.1016/j.cmet.2020.01.015

[79] Gwinn DM, Shackelford DB, Egan DF, et al. AMPK phosphorylation of raptor mediates a metabolic checkpoint. Mol Cell. 2008;30(2):214–26. 18439900 10.1016/j.molcel.2008.03.003 PMC2674027

[80] Wang D, Xu C, Yang W, et al. E3 ligase RNF167 and deubiquitinase STAMBPL1 modulate mTOR and cancer progression. Mol Cell. 2022;82(4):770-784.e9. 35114100 10.1016/j.molcel.2022.01.002

[81] Martin SB, Reiche WS, Fifelski NA, et al. Leucine and branched-chain amino acid metabolism contribute to the growth of bone sarcomas by regulating AMPK and mTORC1 signaling. Biochem J. 2020;477(9):1579–99. 32297642 10.1042/BCJ20190754

[82] Xue M, Xiao J, Jiang W, et al. Loss of BCAA catabolism enhances Rab1A-mTORC1 signaling activity and promotes tumor proliferation in NSCLC. Transl Oncol. 2023;34:101696. 37216755 10.1016/j.tranon.2023.101696 PMC10209880

[83] Zhang X, Wang X, Yuan Z, et al. Amino acids-Rab1A-mTORC1 signaling controls whole-body glucose homeostasis. Cell Rep. 2021;34(11):108830. 33730578 10.1016/j.celrep.2021.108830 PMC8062038

[84] Wang Y, Luo M, Wang F, et al. AMPK induces degradation of the transcriptional repressor PROX1 impairing branched amino acid metabolism and tumourigenesis. Nat Commun. 2022;13(1):7215. 36433955 10.1038/s41467-022-34747-y PMC9700865

[85] Sun L, Zhang H, Gao P. Metabolic reprogramming and epigenetic modifications on the path to cancer. Protein Cell. 2022;13(12):877–919. 34050894 10.1007/s13238-021-00846-7 PMC9243210

[86] Zhenyukh O, Civantos E, Ruiz-Ortega M, et al. High concentration of branched-chain amino acids promotes oxidative stress, inflammation and migration of human peripheral blood mononuclear cells via mTORC1 activation. Free Radic Biol Med. 2017;104:165–77. 28089725 10.1016/j.freeradbiomed.2017.01.009

[87] White PJ, McGarrah RW, Grimsrud PA, et al. The BCKDH kinase and phosphatase integrate BCAA and lipid metabolism via regulation of ATP-citrate lyase. Cell Metab. 2018;27(6):1281-1293.e7. 29779826 10.1016/j.cmet.2018.04.015 PMC5990471

[88] Abla H, Sollazzo M, Gasparre G, et al. The multifaceted contribution of α-ketoglutarate to tumor progression: an opportunity to exploit? Semin Cell Dev Biol. 2020;98:26–33. 31175937 10.1016/j.semcdb.2019.05.031

[89] Wang X, Chen Y, Wang X, et al. Stem cell factor SOX2 confers ferroptosis resistance in lung cancer via upregulation of SLC7A11. Can Res. 2021;81(20):5217–29. 10.1158/0008-5472.CAN-21-0567 PMC8530936 34385181

[90] Dang L, White DW, Gross S, et al. Cancer-associated IDH1 mutations produce 2-hydroxyglutarate. Nature. 2009;462(7274):739–44. 19935646 10.1038/nature08617 PMC2818760

[91] Lu C, Ward PS, Kapoor GS, et al. IDH mutation impairs histone demethylation and results in a block to cell differentiation. Nature. 2012;483(7390):474–8. 22343901 10.1038/nature10860 PMC3478770

[92] Xu W, Yang H, Liu Y, et al. Oncometabolite 2-hydroxyglutarate is a competitive inhibitor of α-ketoglutarate-dependent dioxygenases. Cancer Cell. 2011;19(1):17–30. 21251613 10.1016/j.ccr.2010.12.014 PMC3229304

[93] Ward PS, Patel J, Wise DR, et al. The common feature of leukemia-associated IDH1 and IDH2 mutations is a neomorphic enzyme activity converting alpha-ketoglutarate to 2-hydroxyglutarate. Cancer Cell. 2010;17(3):225–34. 20171147 10.1016/j.ccr.2010.01.020 PMC2849316

[94] Pan J, Wang Y, Huang S, et al. High expression of BCAT1 sensitizes AML cells to PARP inhibitor by suppressing DNA damage response. J Mol Med (Berl). 2024;102(3):415–33. 38340163 10.1007/s00109-023-02409-1

[95] Carrer A, Trefely S, Zhao S, et al. Acetyl-CoA metabolism supports multistep pancreatic tumorigenesis. Cancer Discov. 2019;9(3):416–35. 30626590 10.1158/2159-8290.CD-18-0567 PMC6643997

[96] Viré E, Brenner C, Deplus R, et al. The Polycomb group protein EZH2 directly controls DNA methylation. Nature. 2006;439(7078):871–4. 16357870 10.1038/nature04431

[97] Wu K, El Zowalaty AE, Sayin VI, et al. The pleiotropic functions of reactive oxygen species in cancer. Nat Cancer. 2024;5(3):384–99. 38531982 10.1038/s43018-024-00738-9

[98] Willson JA, Arienti S, Sadiku P, et al. Neutrophil HIF-1α stabilization is augmented by mitochondrial ROS produced via the glycerol 3-phosphate shuttle. Blood. 2022;139(2):281–6. 34411229 10.1182/blood.2021011010 PMC8832465

[99] Li Z, Gu Z, Wang L, et al. Nuclear translocation of LDHA promotes the catabolism of BCAAs to sustain GBM cell proliferation through the TxN antioxidant pathway. Int J Mol Sci. 2023;24(11):9365. 37298317 10.3390/ijms24119365 PMC10253380

[100] Markowicz-Piasecka M, Huttunen J, Montaser A, et al. Hemocompatible LAT1-inhibitor can induce apoptosis in cancer cells without affecting brain amino acid homeostasis. Apoptosis Int J Program Cell Death. 2020;25(5–6):426–40. 10.1007/s10495-020-01603-7 PMC7244471 32405891

[101] Huttunen KM, Gynther M, Huttunen J, et al. A selective and slowly reversible inhibitor of l-type amino acid transporter 1 (LAT1) potentiates antiproliferative drug efficacy in cancer cells. J Med Chem. 2016;59(12):5740–51. 27253989 10.1021/acs.jmedchem.6b00190

[102] Petersen A-K, Stark K, Musameh MD, et al. Genetic associations with lipoprotein subfractions provide information on their biological nature. Hum Mol Genet. 2012;21(6):1433–43. 22156577 10.1093/hmg/ddr580

[103] Wysong DR, Chakravarty A, Hoar K, et al. The inhibition of Aurora A abrogates the mitotic delay induced by microtubule perturbing agents. Cell Cycle (Georgetown, Tex). 2009;8(6):876–88. 19221504 10.4161/cc.8.6.7897

[104] Badmann S, Mayr D, Schmoeckel E, et al. AKR1C1/2 inhibition by MPA sensitizes platinum resistant ovarian cancer towards carboplatin. Sci Rep. 2022;12(1):1862. 35115586 10.1038/s41598-022-05785-9 PMC8814148

[105] Waickman AT, Powell JD. mTOR, metabolism, and the regulation of T-cell differentiation and function. Immunol Rev. 2012;249(1):43–58. 22889214 10.1111/j.1600-065X.2012.01152.x PMC3419491

[106] Ikeda K, Kinoshita M, Kayama H, et al. Slc3a2 mediates branched-chain amino-acid-dependent maintenance of regulatory T cells. Cell Rep. 2017;21(7):1824–38. 29141216 10.1016/j.celrep.2017.10.082

[107] Cham CM, Gajewski TF. Glucose availability regulates IFN-gamma production and p70S6 kinase activation in CD8+ effector T cells. J Immunol. 2005;174(8):4670–7. 15814691 10.4049/jimmunol.174.8.4670

[108] Boskovic P, Wilke N, Man K-H, et al. Branched-chain amino acid transaminase 1 regulates glioblastoma cell plasticity and contributes to immunosuppression. Neuro Oncol. 2024;26(2):251–65. 37769206 10.1093/neuonc/noad190 PMC10836774

[109] Torigoe M, Maeshima K, Ozaki T, et al. l-Leucine influx through Slc7a5 regulates inflammatory responses of human B cells via mammalian target of rapamycin complex 1 signaling. Mod Rheumatol. 2019;29(5):885–91. 30092695 10.1080/14397595.2018.1510822

[110] Papathanassiu AE, Ko J-H, Imprialou M, et al. BCAT1 controls metabolic reprogramming in activated human macrophages and is associated with inflammatory diseases. Nat Commun. 2017;8:16040. 28699638 10.1038/ncomms16040 PMC5510229

[111] Ko J-H, Olona A, Papathanassiu AE, et al. BCAT1 affects mitochondrial metabolism independently of leucine transamination in activated human macrophages. J Cell Sci. 2020;133(22):jcs247957. 33148611 10.1242/jcs.247957 PMC7116427

[112] Soares JDP, Howell SL, Teixeira FJ, et al. Dietary amino acids and immunonutrition supplementation in cancer-induced skeletal muscle mass depletion: a mini-review. Curr Pharm Des. 2020;26(9):970–8. 32067606 10.2174/1381612826666200218100420

[113] Tada T, Kumada T, Toyoda H, et al. Oral supplementation with branched-chain amino acid granules prevents hepatocarcinogenesis in patients with hepatitis C-related cirrhosis: a propensity score analysis. Hepatol Res. 2014;44(3):288–95. 23607436 10.1111/hepr.12120

[114] Nojiri S, Fujiwara K, Shinkai N, et al. Effects of branched-chain amino acid supplementation after radiofrequency ablation for hepatocellular carcinoma: a randomized trial. Nutrition (Burbank, Los Angeles County, Calif). 2017;33:20–7. 27908546 10.1016/j.nut.2016.07.013

[115] Imanaka K, Ohkawa K, Tatsumi T, et al. Impact of branched-chain amino acid supplementation on survival in patients with advanced hepatocellular carcinoma treated with sorafenib: a multicenter retrospective cohort study. Hepatol Res. 2016;46(10):1002–10. 26690886 10.1111/hepr.12640

[116] Huang L, Li G, Zhang Y, et al. Small-molecule targeting BCAT1-mediated BCAA metabolism inhibits the activation of SHOC2-RAS-ERK to induce apoptosis of triple-negative breast cancer cells. J Adv Res. 2024;28:S2090-1232(24)00476-4.10.1016/j.jare.2024.10.02139490614

[117] Zhang T, Pan Z, Gao J, et al. Branched-chain amino acid transaminase 1 confers EGFR-TKI resistance through epigenetic glycolytic activation. Signal Transduct Target Ther. 2024;9(1):216. 39143065 10.1038/s41392-024-01928-8 PMC11324870

[118] Cai Z, Chen J, Yu Z, et al. BCAT2 shapes a noninflamed tumor microenvironment and induces resistance to anti-PD-1/PD-L1 immunotherapy by negatively regulating proinflammatory chemokines and anticancer immunity. Adv Sci (Weinheim, Baden-Wurttemberg, Germany). 2023;10(8):e2207155. 10.1002/advs.202207155 PMC10015882 36642843

[119] Deng H, Zhou J, Sundersingh F, et al. Discovery and Optimization of Potent, Selective, and in Vivo Efficacious 2-Aryl Benzimidazole BCATm Inhibitors. ACS Med Chem Lett. 2016;7(4):379–84. 27096045 10.1021/acsmedchemlett.5b00389 PMC4834658

[120] Vander Heiden MG, DeBerardinis RJ. Understanding the intersections between metabolism and cancer biology. Cell. 2017;168(4):657–69. 28187287 10.1016/j.cell.2016.12.039 PMC5329766

[121] Cluntun AA, Lukey MJ, Cerione RA, et al. Glutamine metabolism in cancer: understanding the heterogeneity. Trends Cancer. 2017;3(3):169–80. 28393116 10.1016/j.trecan.2017.01.005 PMC5383348

[122] Xu E, Ji B, Jin K, et al. Branched-chain amino acids catabolism and cancer progression: focus on therapeutic interventions. Front Oncol. 2023;13:1220638. 37637065 10.3389/fonc.2023.1220638 PMC10448767

[123] Peng H, Wang Y, Luo W. Multifaceted role of branched-chain amino acid metabolism in cancer. Oncogene. 2020;39(44):6747–56. 32978521 10.1038/s41388-020-01480-z PMC7606751

[124] Dimou A, Tsimihodimos V, Bairaktari E. The critical role of the branched chain amino acids (BCAAs) catabolism-regulating enzymes, branched-chain aminotransferase (BCAT) and branched-chain α-keto acid dehydrogenase (BCKD), in human pathophysiology. Int J Mol Sci. 2022;23(7):4022. 35409380 10.3390/ijms23074022 PMC8999875

[125] Wang J, Wang W, Zhu F, et al. The role of branched chain amino acids metabolic disorders in tumorigenesis and progression. Biomed Pharmacother. 2022;153:113390. 36076478 10.1016/j.biopha.2022.113390

[126] Plaks V, Kong N, Werb Z. The cancer stem cell niche: how essential is the niche in regulating stemness of tumor cells? Cell Stem Cell. 2015;16(3):225–38. 25748930 10.1016/j.stem.2015.02.015 PMC4355577

[127] Ma N, Chen X, Johnston LJ, et al. Gut microbiota-stem cell niche crosstalk: a new territory for maintaining intestinal homeostasis. iMeta. 2022;1(4):e54. 38867904 10.1002/imt2.54 PMC10989768

